# Promoter-level transcriptome in primary lesions of endometrial cancer identified biomarkers associated with lymph node metastasis

**DOI:** 10.1038/s41598-017-14418-5

**Published:** 2017-10-26

**Authors:** Emiko Yoshida, Yasuhisa Terao, Noriko Hayashi, Kaoru Mogushi, Atsushi Arakawa, Yuji Tanaka, Yosuke Ito, Hiroko Ohmiya, Yoshihide Hayashizaki, Satoru Takeda, Masayoshi Itoh, Hideya Kawaji

**Affiliations:** 1 Department of Obstetrics & Gynecology, Juntendo University Faculty of Medicine, Tokyo, Japan; 2 Division of Genomic Technologies, RIKEN Center for Life Science Technologies, Yokohama, Japan; 3 Intractable Disease Research Center, Juntendo University Graduate School of Medicine, Tokyo, Japan; 4 Department of Human Pathology, Juntendo University Faculty of Medicine, Tokyo, Japan; 5 Preventive Medicine and Applied Genomics Unit, RIKEN Advanced Center for Computing and Communication, Yokohama, Japan; 6 RIKEN Preventive Medicine and Diagnosis Innovation Program, Wako, Japan

## Abstract

For endometrial cancer patients, lymphadenectomy is recommended to exclude rarely metastasized cancer cells. This procedure is performed even in patients with low risk of recurrence despite the risk of complications such as lymphedema. A method to accurately identify cases with no lymph node metastases (LN−) before lymphadenectomy is therefore highly required. We approached this clinical problem by examining primary lesions of endometrial cancers with CAGE (Cap Analysis Gene Expression), which quantifies promoter-level expression across the genome. Fourteen profiles delineated distinct transcriptional networks between LN + and LN− cases, within those classified as having the low or intermediate risk of recurrence. Subsequent quantitative reverse transcription polymerase chain reaction (qRT-PCR) analyses of 115 primary tumors showed *SEMA3D* mRNA and *TACC2* isoforms expressed through a novel promoter as promising biomarkers with high accuracy (area under the receiver operating characteristic curve, 0.929) when used in combination. Our high-resolution transcriptome provided evidence of distinct molecular profiles underlying LN + /LN− status in endometrial cancers, raising the possibility of preoperative diagnosis to reduce unnecessary operations in patients with minimum recurrence risk.

## Introduction

In developed countries, endometrial cancer is the most common type of cancer of the female reproductive tract, and its incidence has been increasing in recent years^1,2^. Lymph node assessment is critically important to decide treatment in endometrial carcinoma. If systematic pelvic lymphadenectomy is performed, about 12–13% of patients with clinically uterine-confined endometrial cancer have positive lymph nodes^3^.

According to the National Comprehensive Cancer Network (NCCN) guidelines, lymphadenectomy is recommended for adequate staging, and should be performed based on individual considerations of the risk.

Therefore, a practicable and accurate method to assess lymph node metastasis without lymphadenectomy is required, particularly for cases of low risk of recurrence. Tumor marker carbohydrate antigen 125 (CA-125) in the blood is frequently elevated in patients at an advanced stage with a high tumor volume, but is not associated with lymph node metastasis at the early stage^4^. Imaging-based diagnostic tests, such as computed tomography (CT), magnetic resonance imaging (MRI), and positron emission tomography (PET), are not sensitive enough to detect micrometastases^5,6^. Histological grade, myometrial invasion, tumor diameter ≥ 2 cm, and extrauterine disease are risk factors for lymph node metastasis^7–9^; however, preoperative and intraoperative diagnosis using these factors is inaccurate and inadequate for clinical decision making^10,11^. Sentinel lymph node (SLN) mapping was expected to be accurate, as demonstrated for breast cancer^12^, but the lymphatic drainage patterns of the uterus are more complicated than those of the breast. The recent FIRES trial^13^ examined the sensitivity and negative predictive value of SLN mapping, in comparison with the gold standard of complete lymphadenectomy, for detection of metastatic lymph nodes of endometrial cancer. In this trial, 18 gynecology oncologists without prior SLN biopsy experience demonstrated a > 99% negative predictive value of negative SLN biopsy and a 3% rate of missing lymph node dissection if SLN biopsy were relied upon without systematic lymphadenectomy^13^.

This result shows that SLN mapping can circumvent the need for skilled SLN biopsy experience. The current NCCN guidelines have endorsed SLN mapping as a technique for the staging of endometrial cancer, with a level 2B category of evidence and consensus. SLN mapping may be considered in select patients for the surgical staging of apparent uterine-confined malignancy when there is no metastasis demonstrated by imaging studies and no obvious extrauterine disease at exploration.

An alternative approach is to conduct a molecular analysis of primary cancer cells derived from patients, since the cancer cells in the primary tumor have to be dissected regardless of LN + /LN− status. Multi-omics analyses across the genome, such as somatic mutation and gene expression analyses, have been applied to various cancer types, and have effectively defined molecular-based subtypes^14–16^, including in endometrial cancer^17^; however, classifying patients by clinical factors remains challenging. In particular, discrimination of LN + /LN− status in patients with low-risk of recurrence has not been elucidated to date.

In recent studies, we built an atlas of human cellular states based on activities of regulatory elements across the genome, such as promoters^18^ and enhancers^19^, by monitoring transcription initiation activities with CAGE (Cap Analysis of Gene Expression)^20^. The method determines 5′-end sequences of messenger RNA and long noncoding RNAs by using next-generation sequencers, where complementary DNAs (cDNAs) are synthesized from RNA extracts, cDNAs corresponding to RNA 5′-ends are selected by using the cap-trapper method^21^ and sequenced. Obtained reads are aligned with the genome sequences and their 5′-ends indicate frequencies of transcription initiation at single-base resolution^22^. Here we examined the ability of this technology to discriminate patients based on LN + /LN− status. We further explored the data to identify marker molecules that classify the two groups of patients, and conducted quantitative reverse transcription polymerase chain reaction (qRT-PCR) analysis to examine each marker’s utility and performance.

## Results

### Collection of uterine cancer tissues and definition of risk groups

Uterine cancer tissues were obtained from surgically-extracted uterus of 121 patients for this study. Patients with sarcomatoid histology or treated with neoadjuvant chemotherapy (NAC) were excluded, resulting in 115 selected patients of which 15.7% had lymph node metastasis. The mean age at diagnosis was 56 years. Most patients were diagnosed as FIGO (International Federation of Gynecology and Obstetrics) stage I. Of the total patients, 90.4% were diagnosed with endometrioid adenocarcinoma, including Grade 1 (67.0%), Grade 2 (25.2%), and Grade 3 (7.8%). Cases with non-endometrioid histological type included the following: 4 clear cell carcinoma, 2 serous adenocarcinoma, 1 endometrioid and clear cell carcinoma, 1 endometrioid and serous adenocarcinoma, 1 small cell carcinoma, 1 adenosquamous carcinoma and 1 clear cell and serous adenocarcinoma. The clinical characteristics of the patients are listed in Table 1.

**Table 1:** Characteristics of the 115 uterine cancer patients.

**Characteristics**	**n = 115**	**%**
Age mean (range)	56 (31–85)	—
Gravida median (range)	1 (0–5)	—
Parity median (range)	0 (0–4)	—
Menopause	64	55.7
Body mass index (BMI)> 30	19	16.5
Diabetes mellitus	17	14.8
Hypertension	23	20.0
Currently smoker	10	8.7
History of breast cancer	11	9.6
History of other adenocarcinoma	8	7.0
Histologic type^a^		
Endometrioid	104	90.4
Nonendometrioid	11	9.6
FIGO stage^b^		
I	84	73.0
II	6	5.2
III	17	14.8
IV	8	0.0
Histologic differentiation		
Grade 1	77	67.0
Grade 2	29	25,2
Grade 3	9	7.8
Lymph node metastasis		
Negative	97	84.3
Positive	18	15.7

NOTE: Patients with sarcomatoid histology or those treated with NAC (neoadjuvant chemotherapy) were excluded. ^a^Nonendometrioid: 4 clear cell, 2 serous, 2 mixed epithelial tumors (1 endometrioid and clear cell, 1 endometrioid and serous), 1 small cell, 1 adenosquamous cancer, 1 clear cell and serous. ^b^Patients diagnosed using the International Federation of Gynecology and Obstetrics (FIGO) 1988 classification were re-classified according to the FIGO 2008 classification.

The recurrence risks of the patients were assessed based on the histology and tumor progression (i.e., confined to uterine corpus or not), rather than lymph node metastasis. Endometrioid adenocarcinoma grade 1 or 2 with neither cervical stromal invasion nor progression to extra-uterine lesion was considered as low risk of recurrence, endometrioid adenocarcinoma grade 3 with less than one-half myometrial invasion was considered intermediate risk, and remaining types were considered high risk. The numbers of recruited patients with each level of recurrence risk are listed in Supplementary Table 1. Two groups of patients with low-intermediate risk and high risk were examined separately hereafter.

Table 2 shows the clinicopathological features of the patients included in the study. The mean age at diagnosis was 58 and 56 years for patients with and without lymph node metastasis, respectively. The percentage of postmenopausal women was not significantly different between the LN + and LN− groups. Previous studies reported that histological grade, tumor diameter ≥ 2 cm, myometrial invasion, cervical stromal invasion, and lymph-vascular invasion are independent risk factors for lymph node metastasis^9^. We found that myometrial invasion, lymph-vascular invasion and the defined recurrence risks differed significantly according to LN + /LN− status, but not histological grade, tumor diameter ≥ 2 cm, or cervical stromal invasion.

**Table 2:** Clinicopathologic features of patients with and without lymph node metastasis.

Variable	Lymph node metastasis		***P value***
	Negative	Positive	
	**(n = 97)**	**(n = 18)**	
Age mean (range)	56 (41–76)	58 (31–85)	0.474
Menopause	53 (54.6%)	11 (61.1%)	0.797
Histologic type			0.909
Endometrioid Grade 1	66 (68.0%)	12 (66.7%)	
Endometrioid Grade 2	15 (15.5%)	2 (11.1%)	
Endometrioid Grade 3	7 (7.2%)	2 (11.1%)	
Nonendometrioid	9 (9.3%)	2 (11.1%)	
Tumor size^a^			0.237
≤ 2 cm	24 (24.7%)	2 (11.1%)	
> 2 cm	73 (75.3%)	16 (88.9%)	
Myometrial invasion^b^			0.012
≤ 1/2	70 (72.2%)	7 (38.9%)	
> 1/2	27 (27.8%)	11 (61.1%)	
Cervical stromal invasion			0.332
Present	5 (5.2%)	2 (11.1%)	
Absent	92 (94.8%)	16 (88.9%)	
Lymph-vascular invasion			< 0.001
Present	13 (13.4%)	13 (72.2%)	
Absent	84 (86.6%)	5 (27.8%)	
Risk of recurrence			< 0.01
low-intermediate risk	77 (90.6%)	8 (9.4%)	
high risk	20 (66.7%)	10 (33.3%)	

NOTE: ^a^Tumor size was classified based on maximum tumor dimension. ^b^Depth of myometrial invasion was divided into two categories: ≤ 1/2 or > 1/2 of width of muscle layer. ^c^Statistical analysis was performed using the Mann–Whitney test and Fisher’s exact tests, where appropriate. Significant correlations are marked in bold.

### Distinct transcription networks in cancer cells associated with LN + /LN− status in patients with low-intermediate risk of recurrence

**Figure 1:**
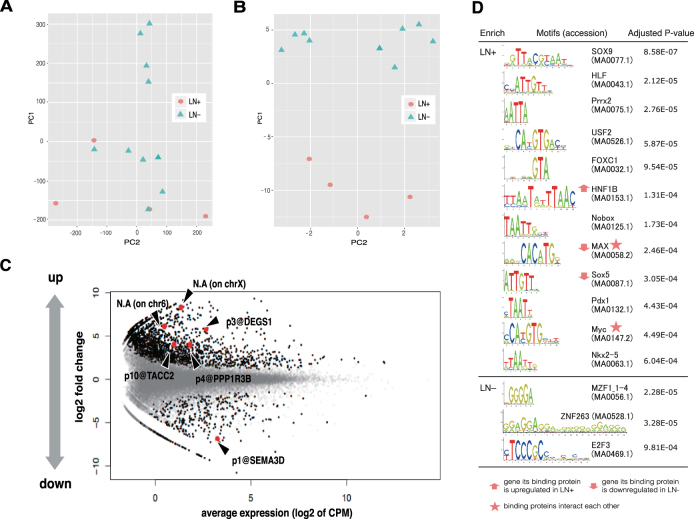
Promoter-level expression analyses of endometrial cancer tissues with LN + and LN− status (**A,B**). Plots of principal components analysis based on expression of the entire promoter set (**A**), and identified marker candidates (**B**), where X- and Y- axis represent first and second principal components respectively. (**C**) MA-plot of the monitored expressions of individual promoters. Non-differentially expressed (gray dots), differentially expressed (black dots), and the six selected candidates of biomarkers (red dots) are shown. (**D**) TFBS (transcription factor binding site) motifs enriched in the differentially activated promoters.

We applied CAGE, a method quantifying activities of individual promoters across the genome, to uterine cancer tissue from 15 of the 85 low-intermediate risk patients; these consisted of five cases of LN + and ten cases of LN−; 14 of the 15 profiles were selected as good quality and used for subsequent analysis (see Materials and Methods). In principal component analysis based on the expression of the entire genome-wide promoter set (Fig. 1A,B), the cancer cells showed different tendencies depending on the LN + /LN− status of the patients but the distinction was not clear.

Differential analysis with a false discovery rate (FDR) of < 5% (Fig. 1C) resulted in the identification of 1,862 and 408 promoters that were upregulated and downregulated, respectively, in LN + cases. Within the up-regulated promoters in LN + , we found enrichment of gene ontology (GO) terms related to keratinization (Table 3), which indicates a squamous cell signature of epithelial cells. We also examined enrichment of transcription factor-binding motifs^23,24^ in the vicinity of the promoters (Fig. 1D), and found three differentially expressed genes encoding proteins that bind the enriched motifs: *HNF1B* (HNF1 homeobox B) was up-regulated in LN + , and *SOX5* (SRY-box 5) and *MAX* (MYC associated factor X) were down-regulated in LN + , and their binding motifs were enriched in up-regulated promoters. Down-regulation of *SOX5* and *MAX* in LN + tumors is consistent with the enrichment of their binding motifs when their mode of action is considered: SOX5 is reported as predominantly a repressor^25^ and MAX is able to repress MYC activity although it has a dual function^26^. These results indicate that endometrial cancer cells of LN + patients are likely controlled by transcriptional regulatory networks that are distinct from those in LN− patients.

**Table 3:** Enrichment of gene ontology (GO) terms within promoters up- and down- regulated in LN + tissues.

**Upregulated**	**Term ID**	**Term**	**ont**	**N**	**DE**	**P.DE**	**FDR.DE**
LN−	GO:0043588	skin development	BP	186	33	7.49E–10	1.46E–05
	GO:0060429	epithelium development	BP	882	88	4.39E–09	8.55E–05
	GO:1903522	regulation of blood circulation	BP	173	30	7.74E–09	0.000150795
	GO:0044057	regulation of system process	BP	319	43	1.47E–08	0.00028678
	GO:0030855	epithelial cell differentiation	BP	464	55	1.52E–08	0.000295929
	GO:0031424	keratinization	BP	21	10	3.38E–08	0.000657674
	GO:0008544	epidermis development	BP	234	34	7.82E–08	0.001522396
	GO:0030216	keratinocyte differentiation	BP	81	18	1.71E–07	0.003323597
	GO:0048878	chemical homeostasis	BP	784	76	1.80E–07	0.00350935
	GO:0008015	blood circulation	BP	325	41	2.04E–07	0.003975223
	GO:0042060	wound healing	BP	595	62	2.22E–07	0.004319577
	GO:0003013	circulatory system process	BP	327	41	2.41E–07	0.004698017
	GO:0050877	neurological system process	BP	557	59	2.53E–07	0.004926144
	GO:0050832	defense response to fungus	BP	20	9	3.09E–07	0.006008942
	GO:0018149	peptide cross-linking	BP	26	10	3.99E–07	0.007766896
LN−	GO:0007610	Behavior	BP	537	27	6.06E–08	0.001182003

NOTE: Within the result of GO enrichment analysis by DAVID, only terms that are classified as biological process (BP) and are associated with less than one thousand genes were shown.

### LN + /LN− marker candidates

**Figure 2:**
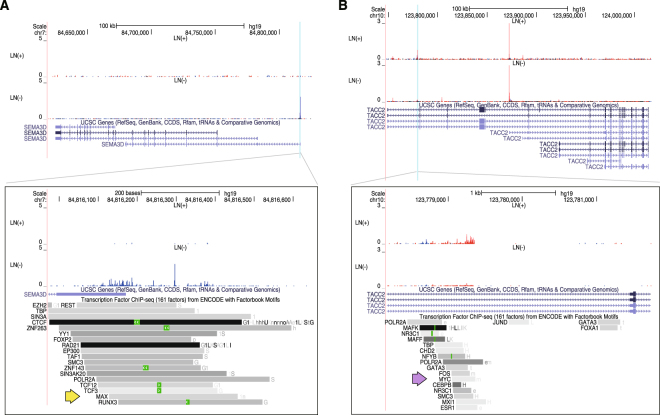
Promoter activities of *SEMA3D* and *TACC2*. Genomic view of gene structure of *SEMA3D* (**A**) and *TACC2* (**B**), and averaged signal of transcription initiation activities (red and blue indicate transcription in forward and reverse strand) in primary endometrial cancer from patients with LN + or LN− status. The upper panels show genomic views of the gene structures, with candidate promoters highlighted as sky blue. The lower panels show close-up views of the candidate promoters with ChIP-seq peaks identified by the ENCODE project (Mar 2012 Freeze; all 690 TF ChIP-seq data passing quality assessment; green boxes indicate sequences that match motifs in Factorbook^28^). Yellow and purple arrows indicate peaks identified by ChIP-seq by using antibodies agains MAX and MYC respectively. Genomic views of the other candidates are shown in Supplementary Figure 2.

We further narrowed down the list of differentially expressed promoters (2,270 promoters in total, including both the upregulated and downregulated promoters described above) by using the following criteria: (i) a strict cutoff of statistical significance in differential analysis (FDR < 0.01%), (ii) a certain level of expression (median expression of > 4 cpm [counts per million] in at least one group) to focus on detectable targets with qRT-PCR, (iii) a substantial difference in expression level between the two groups (more than 16-fold difference in group means), and (iv) a high prediction performance of the two groups by ROC analysis (AUC > 95%). This resulted in six candidates (Fig. 1C, red dots; Supplementary Table 2; Supplementary Figure 1), four of which were annotated as promoters of protein coding genes. No associated genes were found for the remaining two candidates. We manually inspected transcription factor binding sites experimentally identified by the ENCODE project^27,28^, and found evidence of MAX binding to *SEMA3D* (Semaphorin 3D), *DEGS1* (delta 4-desaturase, sphingolipid 1 gene), and *PPP1R3B* (Protein Phosphatase 1 Regulatory Subunit 3B) loci, in particular the promoters defined by the FANTOM5 project as p1@SEMA3D, p3@DEGS1, and p4@PPP1R3B. We also found evidence of MYC binding to *TACC2* (transforming acidic coiled-coil containing protein 2) promoters (p10@TACC2) (Fig. 2; Supplementary Figure 2). This finding implies that the marker candidates reflect dysregulation of the MYC/MAX network that is tightly associated with LN + /LN− status.

We then focused on the promoters defined as p1@SEMA3D, p3@DEGS1, and p10@TACC2 as validation targets, because there was no information on gene structure available for the non-annotated promoters and an LN− outlier overlapped with the LN + expression range of p4@PPP1R3B.

### Structure of the novel *TACC2* isoforms

**Figure 3:**
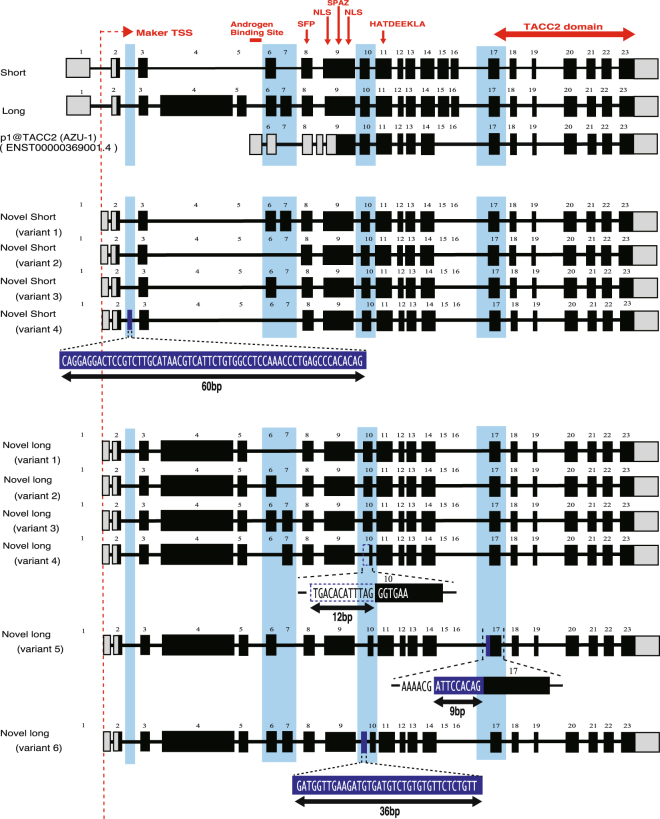
Variant structures of the novel *TACC2* isoforms. Three known isoforms and the novel isoforms of *TACC2* are shown. Exons are depicted as boxes with introns as intervening lines; coding sequences (CDSs) and untranslated regions (UTRs) are depicted as black and gray, respectively. Structural features of TACC2 protein, such as *TACC2* domain, are indicated in red. Variable regions are indicated in light blue. Insertions are indicated with white font with blue background, and deletions are indicated by a dotted line.

Of the three selected metastatic marker candidates, p1@SEMA3D and p3@DEGS1 were located close to the 5′-end of existing gene models (Fig. 2; Supplementary Figures 2 and 3), but no gene models were found with their 5′-end close to p10@TACC2, except for a few expressed sequence tags (ESTs) (Fig. 2). Although *TACC2* mRNAs are reported to have a variety of isoforms^29^, the complete structure of mRNAs expressed via the novel promoter was not clear. Determination of the primary structure of the novel *TACC2* isoforms is required to enable validation by qRT-PCR; therefore, we designed a forward primer for EST (accession BX282179) including the novel transcription start site (TSS) region and a reverse primer for exon 23 (Supplementary Table 3; Supplementary Figure 4). We found two sizes of amplicons (ca. 2 kb and 7.5 kb) by RT-PCR, and sequenced them by primer walking (Supplementary Figure 4). The results indicate that a variety of mRNA isoforms were expressed from the novel TSS, because all of the 10 clones that were sequenced were different from each other. Most of the exons were consistent with existing gene models but their combinations were different (Fig. 3). Exon 6 and exon 7 were skipped depending on the isoforms (this occurred in both of the short and long amplicons), and exon 15 and exon 16 were lacking in all novel isoforms that we sequenced. In one long isoform, a 9-bp sequence was inserted just before exon 17 (Fig. 3).

### Validation of the expressions of *SEMA3D* and the novel *TACC2* isoforms in the group with low-intermediate risk of recurrence

**Figure 4:**
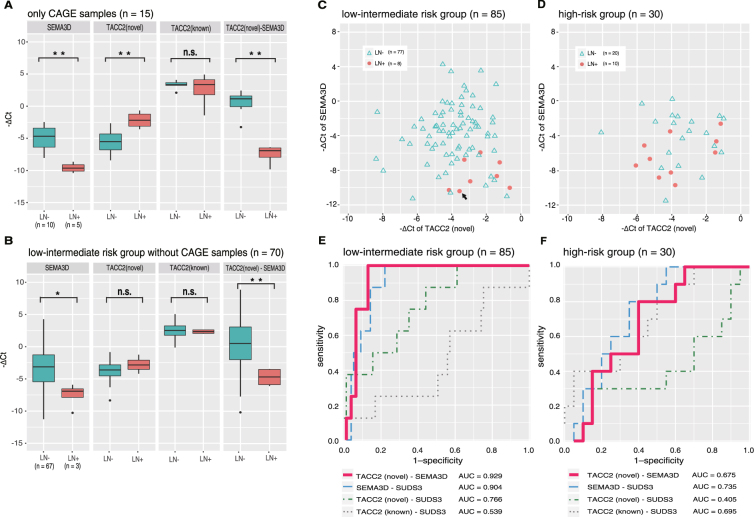
Association of *SEMA3D* mRNA and novel *TACC2* mRNA expression with lymph node metastasis. (**A**) The initial set of cases subjected to CAGE analysis. (**B**) The validation set of cases not assayed by CAGE **P < 0.01; *P < 0.05; n.s., not significant. (**C**) Scatter plots of the low-intermediate risk group showing high expression of the novel *TACC2* and *SEMA3D* mRNAs in LN + and in LN− respectively. The Ct values of one of the LN + samples (with RNA less than 2 µg) were calculated by correcting for the quantity of RNA. This sample is indicated by a black arrow. (**D**) For the scatter plot of the high-risk group, a similar trend was observed, but it was not as remarkable as in the low-risk group. (**E**,**F**) ROC analysis of the low-intermediate risk group (**E**) and high-risk group (**F**).

To confirm the expression of the marker candidates with an independent method to CAGE, we employed qRT-PCR using a custom primer pair designed for the novel TACC2 isoforms and pre-designed primers purchased for the remaining targets (see Materials and Methods). We found that expression of *GAPDH* was variable across the uterine cancer tissues, as seen in other tissues^30^, even though its average expression level was not significantly different between LN + and LN− status. There was also no significant difference between endometrioid endometrial cancer (EEC)/non-endometrioid endometrial cancer (NEEC) status. We selected Sin3 histone deacetylase corepressor complex component gene (*SUDS3)* as the control in qRT-PCR analysis since it is more stably expressed gene based on its CAGE profile (see Materials and Methods) (Supplementary Figure 5). Our experiment based on the same 15 patients subjected to the CAGE assay showed significant difference of *SEMA3D’*s expression *(P* = 3.194 × 10^−5^) and the novel isoforms of *TACC2* (*P* = 0.00458) between the LN + and LN−, where Ct values are different as approximately 4.7 (more than 25 fold) and 3.5 (more than 10 fold) on average (Fig. 4A). We found no significant difference in the expression levels of *TACC2*’s known domain (Fig. 4A).

Given their reciprocal expressions, where *SEMA3D* and the novel isoforms of *TACC2* are highly expressed in LN− and LN + respectively, we asked if the difference of their expressions, that is, the relative expression of *SEMA3D* in comparison to the *TACC2*’s novel isoform, could be further significant. We found the relative expression is different with statistical significance (*P* = 1.538 × 10^−5^) and the difference of Ct values as approximately 8.2 (nearly 300 fold) on average. These results successfully confirmed the expression difference identified by CAGE with an independent technology, and suggested the relative expression of *SEMA3D* in comparison to the novel *TACC2*’s isoform is a promising measure to be examined.

### Additional examination of the expressions in distinct sets of patients

Next we examined another set of 70 patients with low-intermediate risk (three cases of LN + and sixty-seven cases of LN−), which were not assayed by CAGE. We found significant difference of the expression of *SEMA3D* (*P* = 0.0150) and its relative expression to the *TACC2*’s novel isoform (*P* = 0.00109), where the differences of Ct values are 4.1 (more than 17 fold) and 0.86 (more than 1.8 fold) on average (Fig. 4B). This confirmed our finding, in particular the association of the relative expression of *SEMA3D* in comparison to the *TACC2*’s novel isoform, in an additional cohort.

We also examined a set of 30 patients with high-risk of recurrence, and found significant difference only in the expression of *SEMA3D* (*P* = 0.00173) (Supplementary Figure 7). This indicates that only the *SEMA3D* expression could be used in the group with high-risk of recurrence, but not for the remainings.

We further examined an additional cohort of patients assayed with RNA-seq by The Cancer Genome Atlas (TCGA). Although RNA-seq is less sensitive than CAGE in monitoring the 5′-ends of most RNA molecules, we found that it was still possible to quantify the novel first exon of *TACC2* with a limited number of reads obtained from the transcripts (ten reads at maximum). Of 548 patients with uterine corpus endometrial carcinoma, 261 with endometrioid adenocarcinoma without clinical stage II, IIB, IIIA IIIB or IV were profiled by single-end RNA-seq. This group of patients was close to the low–intermediate risk group we defined, but included additional patients with endometrioid adenocarcinoma grade 3 with ≥ 50% myometrial invasion. Among the 261 patients, 32 had LN + , 228 had LN−, and one patient was excluded whose clinical stage did not match the number of lymph nodes with metastases. Cumulative distributions of the expression levels of the marker candidates indicated their trends, higher expressions of the novel *TACC2*’s isoforms and *SEMA3D* in LN + and LN− respectively (Supplementary Figure 8A,B). Based on this, we performed Kaplan−Meier analysis of the relative expression of *SEMA3D* in comparison to the *TACC2*’s isoform, and found that the relative expression was associated with a longer recurrence-free survival (*P* = 0.048; Supplementary Figure 8C,D). The significance is quite marginal, but we would like to note that the RNA quantification technology here is less sensitive for quantification of the marker candidates, patient groups are slightly different as inclusion of endometrioid adenocarcinoma grade 3 with ≥ 50% myometrial invasion, and genetic background are different as non-Japanese. While the TCGA cohort is not complete replication to our study, its result has the same trend to our finding, association of the relative expression of *SEMA3D* in comparison to the novel *TACC2*’s isoform with LN−, leading to longer survival.

### Performance assessment of the biomarkers

Next, we asked how well the two biomarkers could discriminate between LN + and LN− based on all 115 patients we recruited. In the case of the low-intermediate risk of recurrence, ROC analysis indicated that the expressions of *SEMA3D* and the novel isoforms of *TACC2* could both discriminate between LN + and LN− (*SEMA3D* AUC, 0.904; novel isoforms of *TACC2* AUC, 0.766; Fig. 4E). In contrast, expression of the known isoforms of *TACC2* had no ability to discriminate (AUC, 0.539). We assessed the statistical significance of the difference between the AUCs by using the bootstrap method (Supplementary Figure 9) and found a substantial difference between *SEMA3D* and the known isoforms of *TACC2* (*P* = 0.002). For the reasons explained in the previous section, *SUDS3* was chosen as an internal control in these experiments; however, we considered measuring only *SEMA3D* and the novel *TACC2* isoform during diagnosis because of their reciprocal expression. We found that the AUC based on the expression difference between *SEMA3D* and the novel *TACC2* isoforms was slightly higher at 0.929 compared with the AUC between *SEMA3D* and *SUDS3* (Fig. 4E), but this difference was not statistically significant (Supplementary Figure 9). These results indicate that the contribution of *SEMA3D* was the most substantial, while the novel isoform of *TACC2* was valuable in combination with *SEMA3D* as additional information or at least as an internal control.

In cases of high risk of recurrence, ROC curves (Fig. 4F) indicate that *SEMA3D* expression discriminated between LN + and LN− status at a moderate level (AUC, 0.735), however the novel isoform of *TACC2* made no contribution.

Optimal cut-off values dividing the cases into the groups with LN + and LN− were determined by maximizing the Youden′s index from the ROC curves based on the expression of SEMA3D and the novel TACC2 isoforms in cases of low-intermediate risk of recurrence. Biomarker based on the expression difference between SEMA3D and the novel TACC2 isoforms had high specificities (87.0%), sensitivities (100%) and negative predict value (100%) at the optimal cut-off point (Supplementary Table 5).

### Localization of *SEMA3D* mRNA and TACC2 protein in uterine cancer tissue

**Figure 5:**
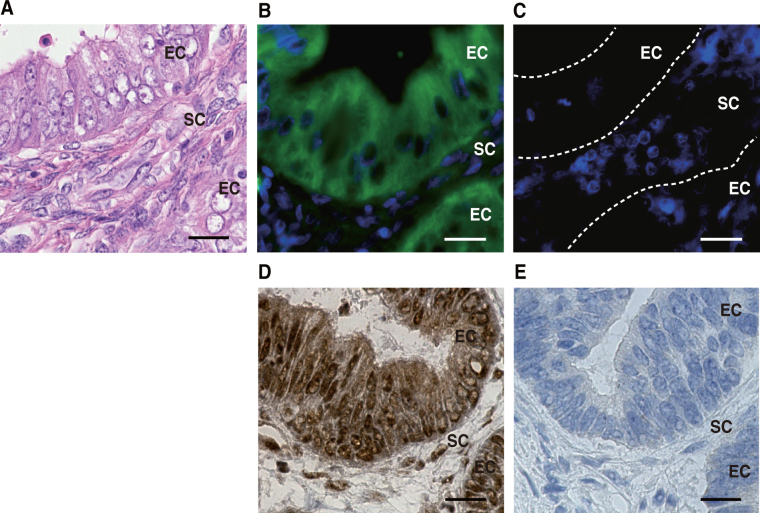
Localization of *SEMA3D* mRNA and TACC2 protein in uterine endometrioid adenocarcinoma cells. (**A**) Hematoxylin and eosin staining. (**B**) *SEMA3D* mRNA *in situ* hybridization assay. *SEMA3D* mRNA (green) and DAPI nuclear stain (blue). (**C**) Negative control for B (control samples were pretreated with RNase A (50 μg/mL) for 30 min at 37 °C prior to the hybridization step). DAPI nuclear stain (blue). (**D**) Immunostaining of TACC2 protein. (**E**) Negative control for D. EC, endometrioid adenocarcinoma cells, SC, stromal cells. Bar, 20 μm.

Last, we asked whether the biomarkers were signatures of cancer cells specifically. Although primary lesions of endometrial cancer were collected for the experiments above, it is possible that non-cancer cells, such as stromal cells, also express the biomarkers. To address this point, we performed immunohistochemical analyses and FISH (fluorescent *in situ* hybridization) in primary tumor specimens. We employed immunostaining of TACC2 protein and found its signal mainly in cancer cells, particularly their nucleus (Fig. 5D,E). We employed FISH for *SEMA3D* mRNA, because the protein product is secreted to the extracellular space^31^, and found *SEMA3D* mRNA signal mainly in the cytoplasm of cancer cells (Fig. 5B,C). These results demonstrate that the identified biomarkers are activated in cancer cells, but not in stromal cells.

## Discussion

In this study, we delineated the transcriptome of primary endometrial cancer tissues with the aim of LN + /LN− discrimination before lymphadenectomy. We first produced a high-resolution transcriptome map reflecting promoter-level expression by using CAGE. The result uncovered differences in transcriptional regulatory networks corresponding to LN + /LN− status within patients with low-intermediate risk of recurrence, that is, endometrioid adenocarcinoma grade 1 or 2 with neither cervical stromal invasion nor progression to extra-uterine lesion. Unexpectedly we found activation of keratinization-related genes in LN + patients, which indicates a squamous cell signature at the molecular level even though the tissue is diagnosed as endometrial adenocarcinoma. Through motif enrichment analysis and differential expression, we also found *HNF1B* to be a potential regulatory factor in LN + patients. A single nucleotide polymorphism in the *HNF1B* locus is reported to be associated with endometrial cancer risk in a European population^32^, and its expression suggests a signature of clear cell carcinoma^33,34^. These discordant signatures can be interpreted as representing atypical adenocarcinoma. In the original histological diagnosis, a squamous signature was found in 3 of 8 LN + cases (37.5%) in the low-intermediate risk group, and in only 7 of 77 LN− cases (9.09%). We revisited the pathology of the eight cases with LN + in the low-intermediate risk group and found a cryptic squamous signature in seven of them (87.5%) consistent with the CAGE result above. We note that cases of squamous differentiation in endometrial adenocarcinomas on average comprise about 25% of cases^35^, and the 85% ratio in the LN + cases is substantially high compared with this average. This implies that a squamous signature might be associated with lymph node metastasis. This notion is consistent with a previous study that reported that squamous metaplasia is correlated with the muscle layer invasion, which may lead to vascular invasion and lymph node metastasis^35^. However, in contrast to adenosquamous carcinoma, a type of endometrial cancer with a squamous signature, adenoacanthoma, has been reported to be associated with a good prognosis^35,36^. This contradiction might be explained by more detailed characterization or subtyping of individual cases at the molecular level; however, it remains to be elucidated.

Furthermore, we found enrichment of MYC and MAX binding motifs in promoters upregulated in LN + cases, which indicates contribution of the MYC/MAX transcriptional regulatory network^26^. As early as 1990, *MYC* was reported to be over-amplified^37^ in endometrial cancer, and its expression and role are still being studied extensively^38,39^. Here, the expression level of *MYC* was not very different between LN + and LN− cases, but the activity of MYC was likely enhanced in LN + cases by depletion of *MAX* and induced epithelial to mesenchymal transition (EMT). Notably, *SOX5*, an EMT inducer through activation of *TWIST1* in breast cancer cells^40–42^, was downregulated in LN + cases. MYC-driven EMT induction is reported as exclusive to SOX5/TWIST1-driven induction in endometriosis^43^, and our result suggests that LN + endometrial carcinoma is driven by MYC-dependent transcription, whereas development of LN− endometrial carcinoma is dependent of SOX5. Taken together, our findings provide the first evidence of distinct subtypes in endometrial carcinoma corresponding to LN + /LN− status in cases with low-intermediate risk of recurrence, where the former represents an atypical state of adenocarcinoma driven by *MYC* and the latter is a benign state of adenocarcinoma.

Based on the distinct transcriptome signatures above, we further explored molecular markers to find those suitable for preoperative diagnosis. We found *SEMA3D* mRNA and novel isoforms of *TACC2* mRNA as potential indicators of LN− and LN + respectively. High-throughput epigenetic profiles^27,28^ indicated MAX and MYC binding to the promoters of *SEMA3D* and *TACC2*, respectively, which is consistent with the contribution of the MYC/MAX network described above. Our follow-up experiments based on qRT-PCR analysis of an expanded group of patients (all levels of risk of recurrence), confirmed substantial association with LN + /LN− status in cases with low-intermediate risk of recurrence, but not in cases with high risk of recurrence. In terms of clinical utilities, importance of preoperative diagnosis is very high for patients of low-intermediate risk of recurrence, but is minor for those with high risk of recurrence, since lymphadenectomy would need to be performed for high-risk cases for its contribution to prognosis^44^, regardless of the presence or absence of lymph node metastasis. From this perspective, the clinical utility of our findings remains very high.

One of the biomarkers of LN− status discovered in the present study, *SEMA3D* mRNA, encodes a protein that is a member of class 3 semaphorins (SEMA3), a secreted protein family consisting of seven members (SEMA3A to SEMA3G) that are involved in cell-cell communication, in particular through interaction with neuropilin and plexin^45^. Other family members are reported to regulate tumor growth and metastasis^46–48^; for example, SEMA3E is reported to be present in cancer cells and to drive metastasis^49^ through the activation of receptor tyrosine kinases, and to have anti-tumorigenesis activity and be down-regulated in advanced tumors^50^. However, SEMA3D has no reports in the context of cancer, except for an indirect suggestion of anti-tumorigenic activity^50^. Because competition between SEMA3D and VEGF leads to inhibition of VEGF-dependent activation of neuropilins^51^, and VEGFC and VEGFD are associated with lymphangiogenesis^52^, we would expect that SEMA3D would play a role in lymphangiogenesis inhibition rather than angiogenesis; however, its precise molecular function in uterine cancer remains to be elucidated.

The other biomarker, a set of novel mRNA isoforms of *TACC2*, has not been described previously. TACC2 is a member of the TACC family, which interacts with centrosome-associated microtubules^53^ that shows aberrations in many tumors. An association between TACC family genes and cancer has been reported: over-amplification of *TACC1* and *TACC2* is observed in breast cancer, and the *TACC3* locus is associated with multiple myeloma^54^; the three family members are characterized by a C-terminal domain (TACC domain)^53^ and are suggested to play distinct roles based on their distinct patterns of centrosome association^55^. A variety of mRNA isoforms of *TACC2* have been reported, including a 3.8-kb isoform termed anti-zuai-1 gene (*AZU-1*), and 4.2-kb and 9.7-kb major isoforms^29^; the isoform *AZU-1* was originally considered a tumor suppressor gene^29^ but recent studies reported increased expression of *TACC2* in breast cancer patients with poor prognosis^56^ and in prostate cancer patients with poor survival rate^57^. However, the role of *TACC2* has never been elucidated in endometrial cancer, and the variety of its isoforms identified in this study has never been exploited in any context. By considering that the isoforms share the same protein coding sequences, one could expect their molecular functions to be identical to those of known isoforms and that elevated TACC2 proteins prompted by the novel isoforms would disrupt microtubule function.

We originally considered using immunohistochemistry to monitor the two biomarkers at the protein level. However, attempts to detect SEMA3D protein did not succeed, likely due to the secretory nature of the SEMA3D protein. Furthermore, because the novel and conventional mRNA isoforms of *TACC2* probably encode the same protein, they would likely be indistinguishable at the protein level. Therefore, quantification of RNA is the most promising approach for these two biomarkers.

We compared the performance of our biomarkers with that of other methods for diagnosing lymph node metastasis, such as SLN mapping and preoperative imaging-based diagnostic tests. Optimal cut-off values for our biomarkers were determined by maximizing the Youden’s index from the ROC curves based on the expression of *SEMA3D* and the novel *TACC2* isoforms in cases of low-intermediate risk of recurrence. Within the low-intermediate risk group, preoperative imaging-based diagnosis (such as CT, MRI, and PET) correctly detected 3 cases of 8 positive lymph node metastasis, whereas, using optimal cut-off values, our biomarkers could detect all 8 cases correctly (Supplementary Table 5). This result underlines the limitation of imaging-based diagnosis. The FIRES trial^13^ examined the performance of SLN mapping in a cohort of 385 cases independently to our study: they reported as 97.2% sensitivity, and 99.6% negative predictive value, which is comparable with the performance of our biomarkers. One clinically important difference between SLN mapping and our approach is the extent of invasion: SLN mapping requires a cervical injection of indocyanine green and excision of the SLN after mapping. The FIRES trial reported that 5.7% (22/385) of patients had serious adverse events, with one related to the study intervention.

Although lymph node assessment based on RNA-level expression in primary endometrial cancer lesions, as proposed in this study, is less invasive than SLN mapping, a number of issues remain to be addressed. The first issue is the delineation of the target isoform of *TACC2* mRNA: the primer pair used for our study captures multiple isoforms, and there is a possibility that only a subset of them are associated with LN + /LN− status. Identification of the best target should increase the performance of the diagnostic test. The second issue is reproducibility of the result in an independent cohort. The number of cases studied here is limited to 85 with low-intermediate risk of recurrence in a total of 115 cases. In an independent cohort (an RNA-seq dataset produced by TCGA) we found an association between longer recurrence-free survival and higher expression levels of *SEMA3D* relative to those of novel *TACC2*′ isoforms. Although the trend is similar to our results, our TCGA-based analysis was limited in the ability to quantify RNA and compatibility of clinical conditions. Multi-institutional validation studies based on larger and compatible groups of patients and the use of a specialized RNA quantification approach, such as qRT-PCR, are crucial. The third issue is the type of specimens required for the diagnostic test. Implementation based on endometrial biopsy or dilatation and curettage should give clinicians sufficient time to consider the need for lymphadenectomy; however, the current study is based on surgically-dissected tumor tissues. The applicability of endometrial biopsy remains to be studied. In the case that only surgically-dissected tumors are suitable, a rapid and accurate method for quantifying the target RNAs needs to be developed so that there is enough time to consider lymphadenectomy.

Although a series of studies remain before the markers detected here can be used in the clinic, our results have opened the door for preoperative diagnosis that minimizes lymphadenectomy in cases with little risk of recurrence.

## Methods

### Patients and sample collection

Patients were recruited from the Department of Obstetrics and Gynecology, Juntendo University Hospital, Tokyo, Japan, from April 2009 to August 2015, following a protocol approved by the ethical review board of Juntendo University Faculty of Medicine (No. 21044). Of the patients that provided written informed consent, 121 patients with uterine cancer were chosen for this study after exclusion of those with sarcomatoid histology or undergoing NAC treatment. The following parameters were obtained from the pathology reports: surgical stages based on the FIGO system 2008 classification, histologic subtype, and grade. Cases originally diagnosed using the FIGO system 1988 classification were re-classified according to the 2008 classification. As part of surgical staging, complete pelvic lymphadenectomy was conducted except for 20 cases. In the exceptional cases, lymphadenectomy was not performed with clinical considerations but no recurrence was found within 2 years; such cases were included as LN− in this study. Lymph node dissection was extended outside the pelvis only if para-aortic lymph nodes metastasis was suspected. After **s**urgical dissection, cancer tissues were obtained from the primary lesion in the extracted uterus and cut into square cubes with approximately 5-mm diameters, frozen immediately in liquid nitrogen, and stored at −80 °C until RNA extraction. Although a larger sample size than the 121 patients recruited here is required for clinical assay development, retrospective validation, and prospective validation (phase 2, 3, 4), this sample size is reasonable for preclinical exploratory (phase 1)^58,59^.

### Ethical considerations

The protocol was approved by the ethical review board of Juntendo University Faculty of Medicine in compliance with the Declaration of Helsinki and current legal regulations in Japan. All patientss provided written informed consent for participation.

### RNA extraction and isolation

Total RNA was extracted and isolated from the 121 samples by using Trizol reagent and the RNeasy Plus Mini Kit (QIAGEN, Valencia, CA, USA). The concentration and A260:A280 ratio of each prepared RNA sample were measured with NanoDrop (Thermo Fisher Scientific, Waltham, MA, USA), and 115 RNA extracts with a A260:A280 ratio of 1.9–2.2 were subjected to subsequent analysis.

### CAGE assay

The CAGE libraries from the purified RNA samples were prepared as described previously^22^. In brief, first strand cDNA synthesis was performed by reverse transcription with SuperScriptIII (Thermo Fisher Scientific), the diols in the cap structure of the ribose sugar and at the 3′ end of the RNA were oxidized with sodium periodate, and then biotinylated with biotin (long arm) hydrazide (Vector Laboratories, Burlingame, CA, USA). Single-stranded RNA portions were digested with RNase ONE (Promega, Fitchburg, Wisconsin, USA), and then biotinylated RNA/cDNA was captured on the surface of Dynabeads M-270 streptavidin (Thermo Fisher Scientific). The cDNA was released by heat denaturation and purified by RNase ONE/H digestion followed by AMPure XP (BioRad, Hercules,CA, USA) purification. The purified single-stranded cDNA was subjected to adaptor ligation at both ends, and the double-stranded cDNA library was created using DeepVent (exo-) DNA polymerase. The generated CAGE cDNA libraries were sequenced by an Illumina HiSeq 2500 sequencer (Illumina, San Diego, CA, USA).

### Computational analysis of CAGE data

CAGE reads including a base ‘N’ or matching a ribosomal RNA sequence (U13369.1) identified by rRNAdust^60^ were discarded. The remaining sequences were aligned to the reference genome (hg19) by using Burrows-Wheeler Aligner^61^, and poorly aligned reads (mapping quality, < 20) were discarded using SAM tools^62^. The robust peak set identified in the FANTOM5 project^18,63^ was used as a reference set for the TSS regions; the number of mapped reads starting from these regions was used as raw signal for the promoter activities. The number of CAGE reads representing promoter activities and the remaining reads are summarized in Supplementary Table 4. We only used data showing more than three million mapped reads, since a low number of reads does not reflect expression levels accurately.

Normalization based on the relative log expression method^64,65^ and differential analyses were conducted using edgeR^65^. Receiver operating characteristic (ROC) curve and area under the ROC curve (AUC) was calculated by using the RCOR package in R^66^.

In motif analysis, genomic DNA sequences in the region form 300-bp upstream to 100-bp downstream of the CAGE peaks defined by the FANTOM5 project were subjected to AME (Analysis of Motif Enrichment) tool^67^, using the motif database of JASPAR CORE 2014 vertebrates^68^. GO enrichment analysis was performed by using DAVID software^69^.

### Cloning and sequencing of novel isoforms TACC2

The novel *TACC2* gene isoforms were amplified by using LA Taq with GC buffer I (Takara Bio Inc., Kusatsu, Japan) with the primers described in Supplementary Table 3 and Supplementary Figure 4. The thermocycling program was performed as follows: an initial cycle at 94 °C for 1 min, followed by 30 cycles of 94 °C for 30 s, 55 °C for 30 s, and 72 °C for 4 min, and a final cycle of 72 °C for 5 min. The amplified fragments were isolated using the PureLink Quick Gel Extraction Kit (Invitrogen Ltd., Paisley, UK), and cloned with TOPO TA Cloning Kits for Subcloning (Invitrogen Ltd.). Briefly, each amplicon was inserted into a plasmid vector (pCR 2.1TM-TOPO vector) and transformed into competent cells (TOP10 cells) according to the manufacturer’s instructions. The entire sequences were determined using the primers listed in Supplementary Table 3, the BigDye Terminator v3.1 Cycle Sequencing kit, and a 3130 Genetic Analyzer (Thermo Fisher Scientific).

### qRT-PCR

Total RNA (2 µg) was reverse-transcribed using the PrimeScript 1st strand cDNA Synthesis Kit (Takara Bio Inc.) and oligo dT primer according to the manufacturer’s instructions. The resulting cDNA was diluted 10-fold with RNase-free water and used as a template for qRT-PCR analysis using a ABI 7500 Fast Real-Time PCR System (Thermo Fisher Scientific) according to the manufacturer’s instructions. For each sample, 10 µL of Fast SYBR Green Master Mix (Thermo Fisher Scientific), 0.4 µL of 10 µM forward primer, 0.4 µL of 10 µM reverse primer, and 2 µL of the 10-fold diluted cDNA solution were used and the solution was made up to 18 µL with DNA- and RNA-free distilled water. The thermocycling program was performed as follows: an initial cycle of 95 °C for 20 s, followed by 40 cycles of 95 °C for 3 s and 60 °C for 30 s. Each reaction was run in triplicate for each template.

The following specific primers were used for *SEMA3D*, *DEGS1*, *SUDS3*, and *TACC2* common domain, (Takara Bio Inc., Primer: HA239516 (forward 5′-CCATCGTTGGGTGCAGTATGA-3′ and reverse 5′-TGCCGCTTTATGAAACTGATGA-3′), HA192490, HA237699, and HA225689, respectively). Custom primers for detecting the novel isoform of *TACC2* and the X chromosome (chrX) were designed by using the Primer-BLAST web tool (http://www.ncbi.nlm.nih.gov/tools/primer-blast/) and were synthesized by Takara Bio Inc; the primer sequences were as follows: novel TACC2 isoform, forward 5′-CCAGTTGCTGAAGGGCAGAA -3′, reverse 5′-GCGGACCTTGGAGTCTGAG -3′ chrX forward, 5′-AAGGCTTGCAGGAAGGTGAA-3′, reverse 5′-TGCACCATCATCCCAACACA-3′.

### Analysis of qRT-PCR data

The following formula was used to calculate the relative amount of the transcripts compared to the internal control: Δ cycle threshold (ΔCt) = Ct of target transcript–Ct of internal control. All Ct values were calculated as the average of triplicates. Differential expression levels between the LN + and LN− patients were evaluated a nonparametric Mann–Whitney U test.

*SUDS3* was used as an internal control instead of *GAPDH* based on the CAGE profile obtained above. Among the genes with a small change of less than two-fold between maximum and minimum expression levels and with a substantial expression of more than 50 cpm, we manually chose a gene with a small standard deviation.

### Analysis of TCGA RNA-seq data

We obtained TCGA clinical records from the GDC Data Portal (https://gdc.nci.nih.gov/), and RNA-seq alignment (BAM) files produced by single-end sequencing were from the GDC Legacy Archive (https://portal.gdc.cancer.gov/legacy-archive/). Only the alignments with mapping quality of >20 were counted by featureCounts^70^ based on Gencode v19^71^ and the genomic coordinates of the novel *TACC2* exon (123,779,113–123,779,400 bp of chr10 in hg19/GRCh37). The read counts per gene were normalized based on the relative log expression method^64,65^ and analyzed for differential expression by using edgeR^65^.

In the Kaplan–Meier analysis, we manually inspected cumulative distributions of expressions in patients with and without lymph node metastasis (Supplementary Figure 8C), and split the patients into two groups, the top 20% and bottom 80% groups according to the relative expression of the novel *TACC2* exon in comparison with *SEMA3D*. This is because of that the difference of the cumulative distributions was maximized with the threshold. Based on the group, survival analysis was performed by using the survival package (https://cran.r-project.org/web/packages/survival/index.html).

### *In-situ* Hybridization

Custom Stellaris FISH (fluorescence *in situ* hybridization) probes were designed against *SEMA3D* mRNA (NM_152754) by utilizing the Stellaris RNA FISH Probe Designer (Biosearch Technologies Inc., Petaluma, CA, USA) available online at www.biosearchtech.com/stellarisdesigner (version #4.1). Formalin-fixed and paraffin-embedded (FFPE) tissue of endometrial cancer was sliced at a thickness of 4 µm by using a microtome, and then mounted onto a microscope slide. The slide-mounted tissue sections were deparaffinized and rehydrated following the manufacturer’s instructions. Sections were incubated in 1 × PBS with 10 μg/mL proteinase K for 20 min at 37 °C. Slide-mounted tissue sections that were used as negative controls were pretreated with 50 μg/mL Ribonuclease A (Nacalai tesque, Kyoto, Japan) for 30 min at 37 °C prior to the hybridization step. Probes were labeled with carboxyfluorescein by following the manufacturer’s instructions (www.biosearchtech.com/stellarisprotocols). The FFPE tissues were hybridized to these custom probes according to the protocol for FFPE tissue; hybridizations were carried out on slides for 16 h at 37 °C and in 200 µL of hybridization solution containing 125 nM probes. DAPI nuclear dye was added during the final wash. Images were captured using an oil-immersion objective with an Axiovert 200 M inverted wide field fluorescence microscope (Zeiss, Oberkochen, Germany).

### Immunohistochemistry

Paraffin-embedded tissues of uterine cancer were sliced at a thickness of 3 µm by using a microtome, and were then deparaffinized. Antigen was retrieved by autoclaving the slides at 120 °C for 10 min in 0.01 M citric acid buffer (pH 6.0). Slides were peroxidase blocked by using 3%(w/v) hydrogen peroxide solution for 10 min, and then incubated in goat serum diluted in PBS containing a 1:2000 dilution of polyclonal TACC2 antibody (Merck Millipore, Darmstadt, Germany, #2397077) overnight at 4 °C. In negative control experiments, normal rabbit IgG (Dako, Glostrup, Denmark, #X0936) was used as the primary antibody. The labeled streptavidin–biotin method was used to add a secondary antibody. The antigen-antibody complex was visualized by using 3,3-diaminobenzidine solution (1 mmol/L 3,3-diaminobenzidine, 50 mmol/L PBS (pH 7.4), and 0.006% H_2_O_2_). Slides were briefly counterstained with hematoxylin.

### Statistical analysis

Differential expression levels between the LN + and LN− patients, monitored by qRT-PCR, were evaluated a nonparametric Mann–Whitney U test. Associations between patients’ clinicopathological characteristics and lymph nodal status were evaluated by using the Fisher’s exact test. The discrimination performances of the biomarker candidates were determined by AUC analysis. The statistical significance of differences between two ROC curves was tested with a bootstrap method with 2,000 replicates in the pROC^72^ package. Statistical tests, calculations of AUC, and production of all graphs were performed using R statistical software version 3.2.2 (http://www.R-project.org). All *P* values described in this study represent two-sided tests. *P* values of less than 0.05 were classed as statistically significant. The Kaplan–Meier estimate and the log-rank test were used to assess the correlations between recurrence-free survival and the expression levels of novel *TACC2* exon and *SEMA3D* and survival in the TCGA Endometrioid Cancer dataset. The survival package was used for the illustration of Kaplan–Meier survival curves and calculation of the *P*-values.

## Electronic supplementary material

supplementary information
