# Supplementary material for: Promoter-level transcriptome in primary lesions of endometrial cancer identified biomarkers associated with lymph node metastasis
Source: Sci Rep. 2017 Oct 26;7:14160. doi: 10.1038/s41598-017-14418-5 (PMC5658375; doi:10.1038/s41598-017-14418-5)
Supplement: Supplementary file 1 — supplementary information [file 41598_2017_14418_MOESM1_ESM.pdf]

Promoter-level transcriptome in primary lesions of endometrial cancer identified biomarkers associated with lymph node metastasis

Emiko Yoshida[1,2], Yasuhisa Terao[1*], Noriko Hayashi[1], Kaoru Mogushi[3], Atsushi Arakawa[4], Yuji Tanaka[2,5], Yosuke Ito[1,5], Hiroko Ohmiya[5], Yoshihide Hayashizaki[6], Satoru Takeda[1], Masayoshi Itoh[2,6], Hideya Kawaji[2,5,6]

[1] Department of Obstetrics & Gynecology, Juntendo University Faculty of Medicine, Japan
[2] Division of Genomic Technologies, RIKEN Center for Life Science Technologies, Japan
[3] Intractable Disease Research Center, Juntendo University Graduate School of Medicine, Japan
[4] Department of Human Pathology, Juntendo University Faculty of Medicine, Japan
[5] Preventive Medicine and Applied Genomics Unit, RIKEN Advanced Center for Computing and Communication, Japan
[6] RIKEN Preventive Medicine and Diagnosis Innovation Program, Japan

」
*Corresponding author:
Yasuhisa Terao
yterao@juntendo.ac.jp
2-1-1 Hongo, Bunkyo, Tokyo 113-8421 Japan
TEL/FAX: +81-3-3813-3111 / +81-3-5689-7460

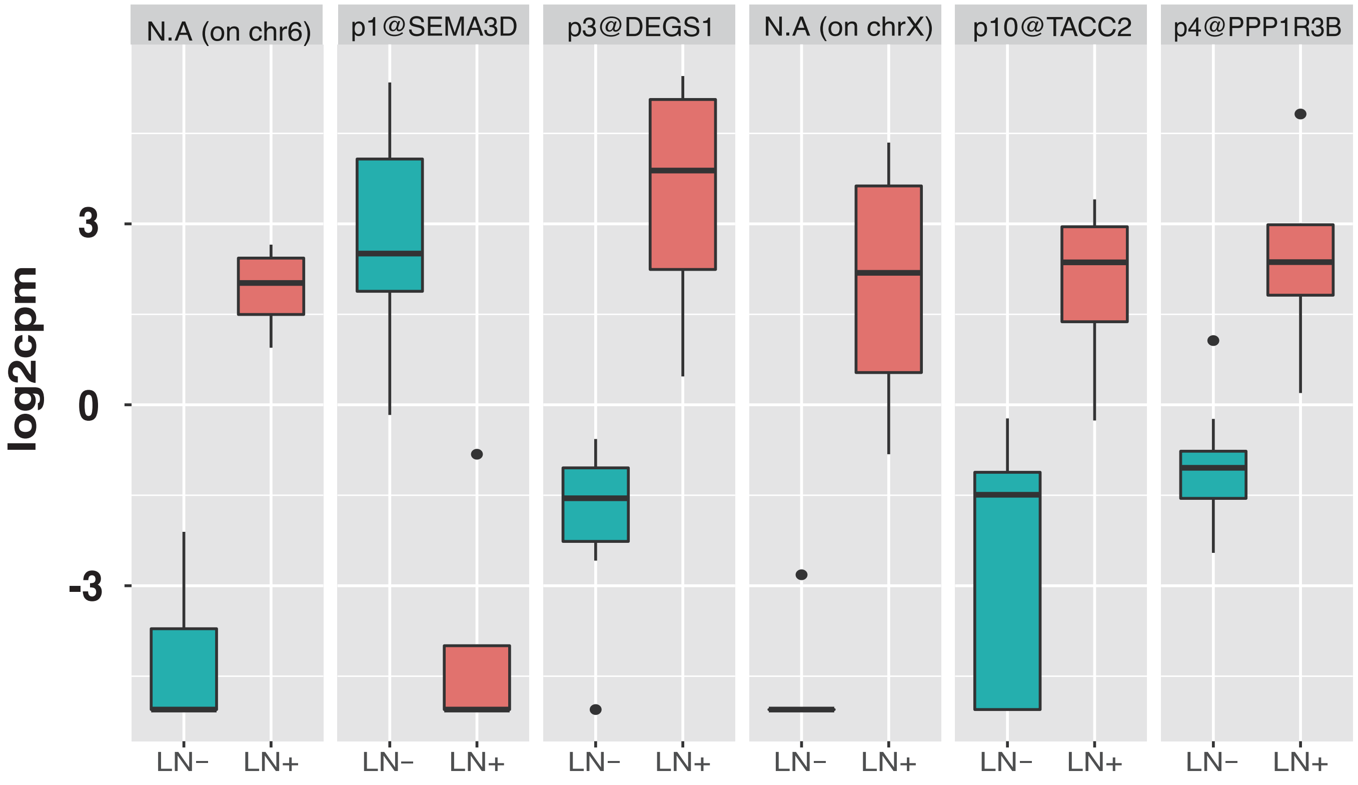

Supplemental Figure 1. Box plots of the candidate expressions within the groups.
N.A. means non-annotated.

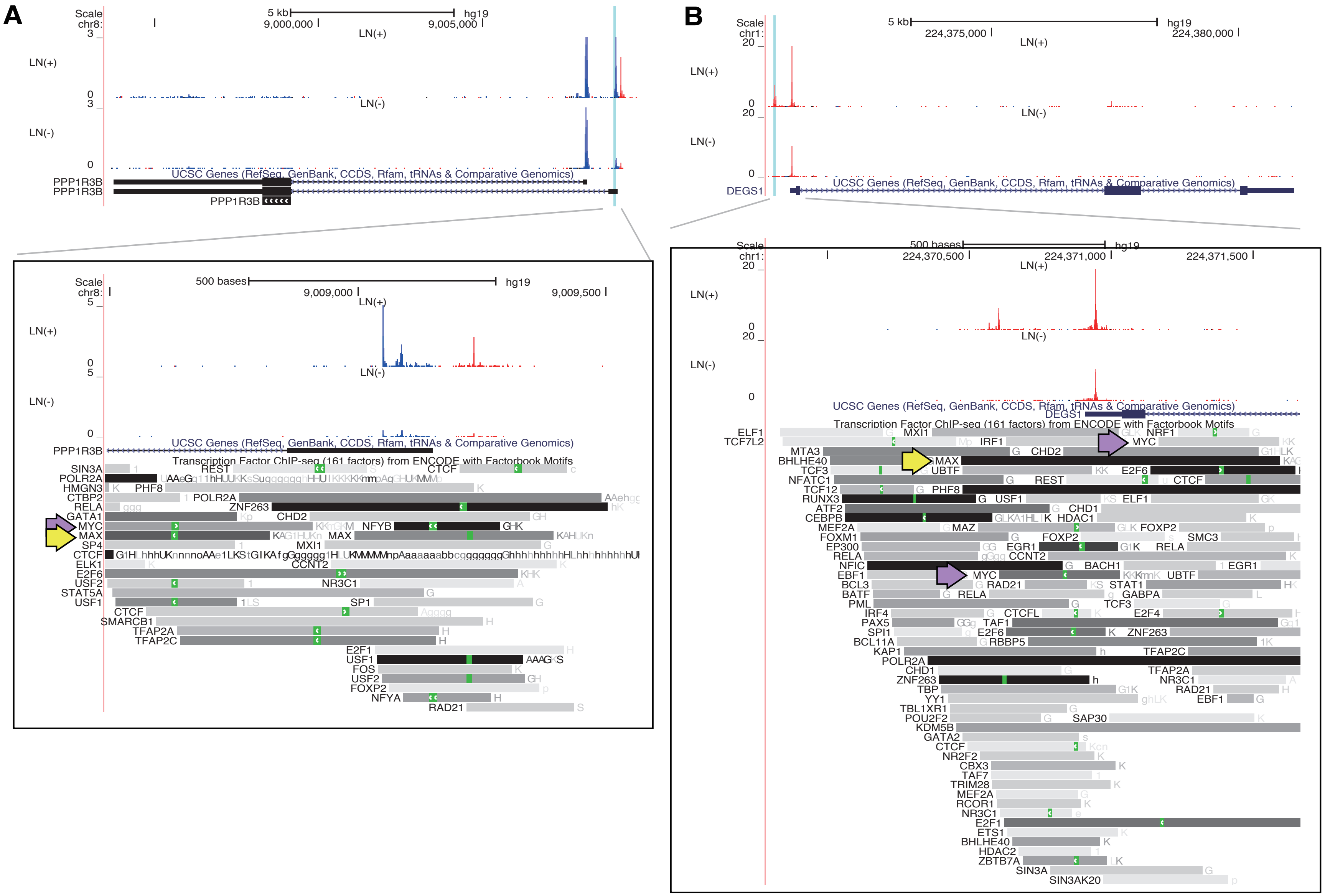

Supplemental Figure 2. Promoter activities of *PPP1R3B* and *DEGS1*.

Genomic view of gene structure of *PPP1R3B* (A) and *DEGS1* (B), and averaged signal of transcription initiation activities (red and blue indicate transcription in forward and reverse strand) in primary endometrial cancer from patients with LN+ or LN– status. The upper panels show genomic views of the gene structures, with candidate promoters highlighted as sky blue. The lower panels show close-up views of the candidate promoters with ChIP-seq peaks identified by the ENCODE project; green boxes indicate motif-matching sequences. Yellow and purple arrows indicate peaks identified by ChIP-seq by using antibody of MAX and MYC respectively.

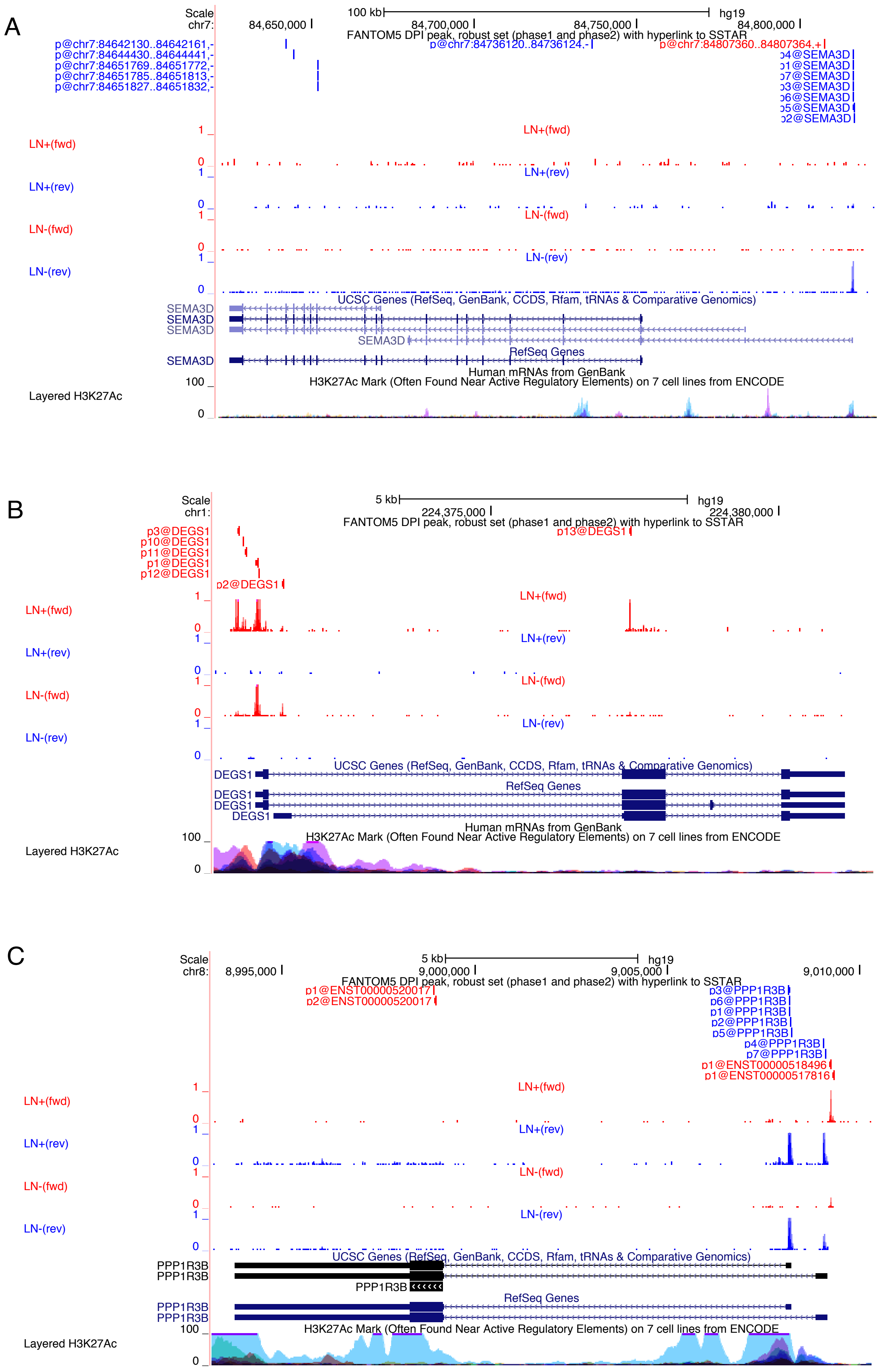

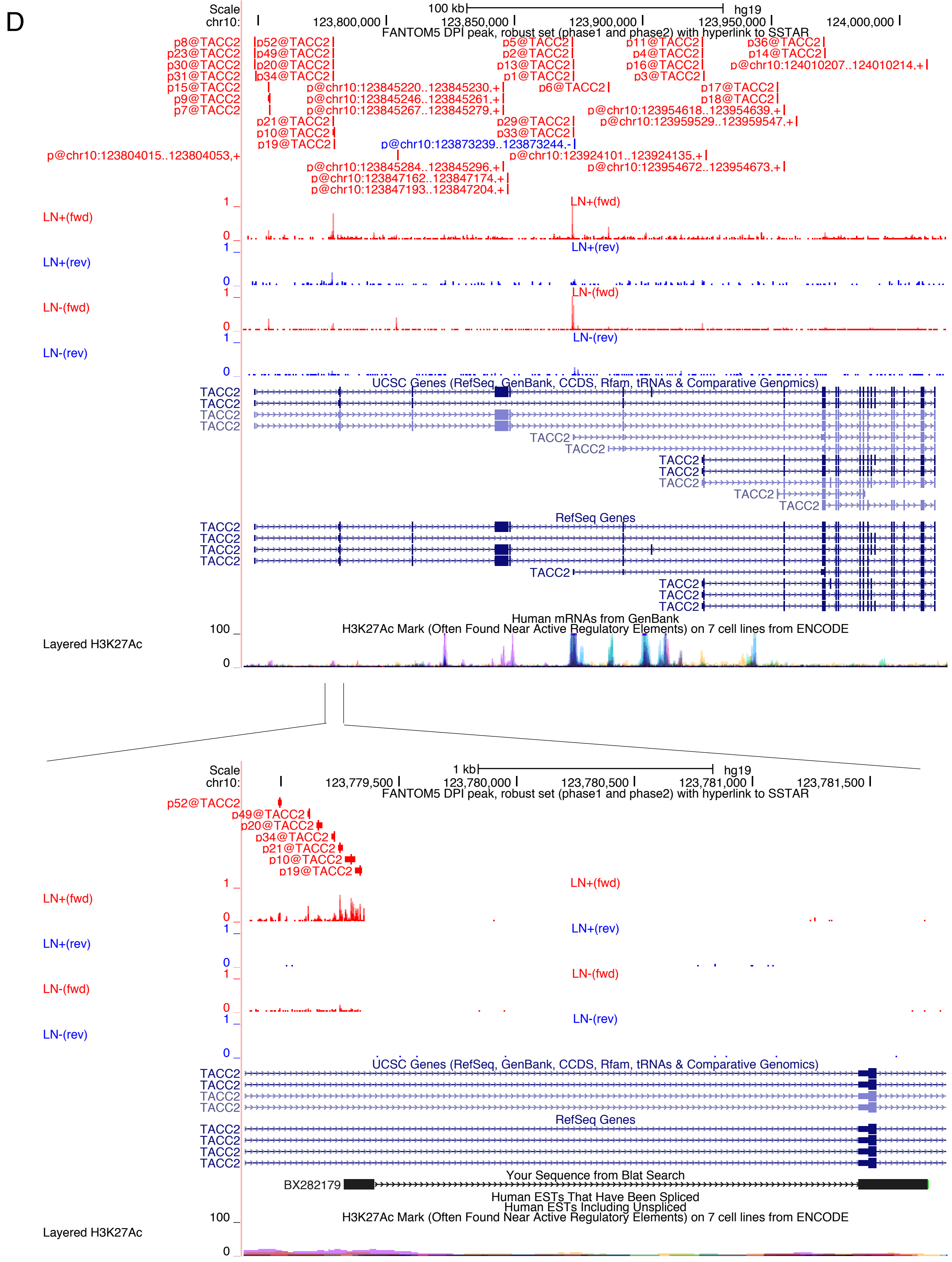

Supplementary Figure 3. Genomic regions analyzed by UCSC Genome Browser.

A. Genomic region of p1@SEMA3D. Blue highlighted background indicates p1@SEMA3D.

B. Genomic region of p3@DEGS1. Blue highlighted background indicates p3@DEGS1.

C. Genomic region of p10@TACC2. The lower panel shows the entire region of the TACC2 locus, and the upper panel is a zoomed view of p10@TACC2. Blue highlighted background indicates p10@TACC2 location in both panels.

D. Genomic region of p4@PPP1R3B. Blue highlighted background indicates p4@PPP1R3B.

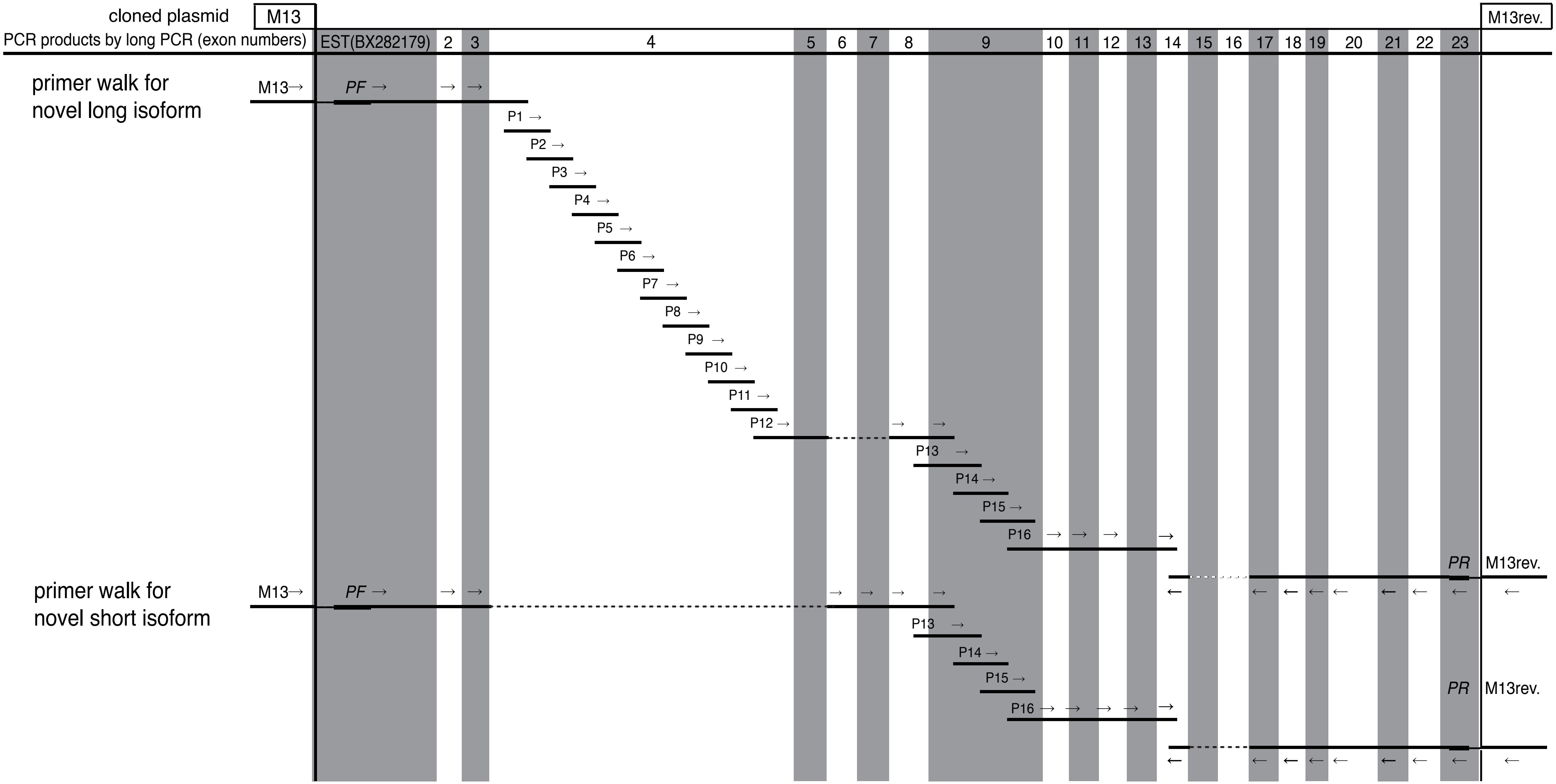

Supplementary Figure 4. Locations of primers for long PCR and primer walk sequence of novel TACC2 isoforms.

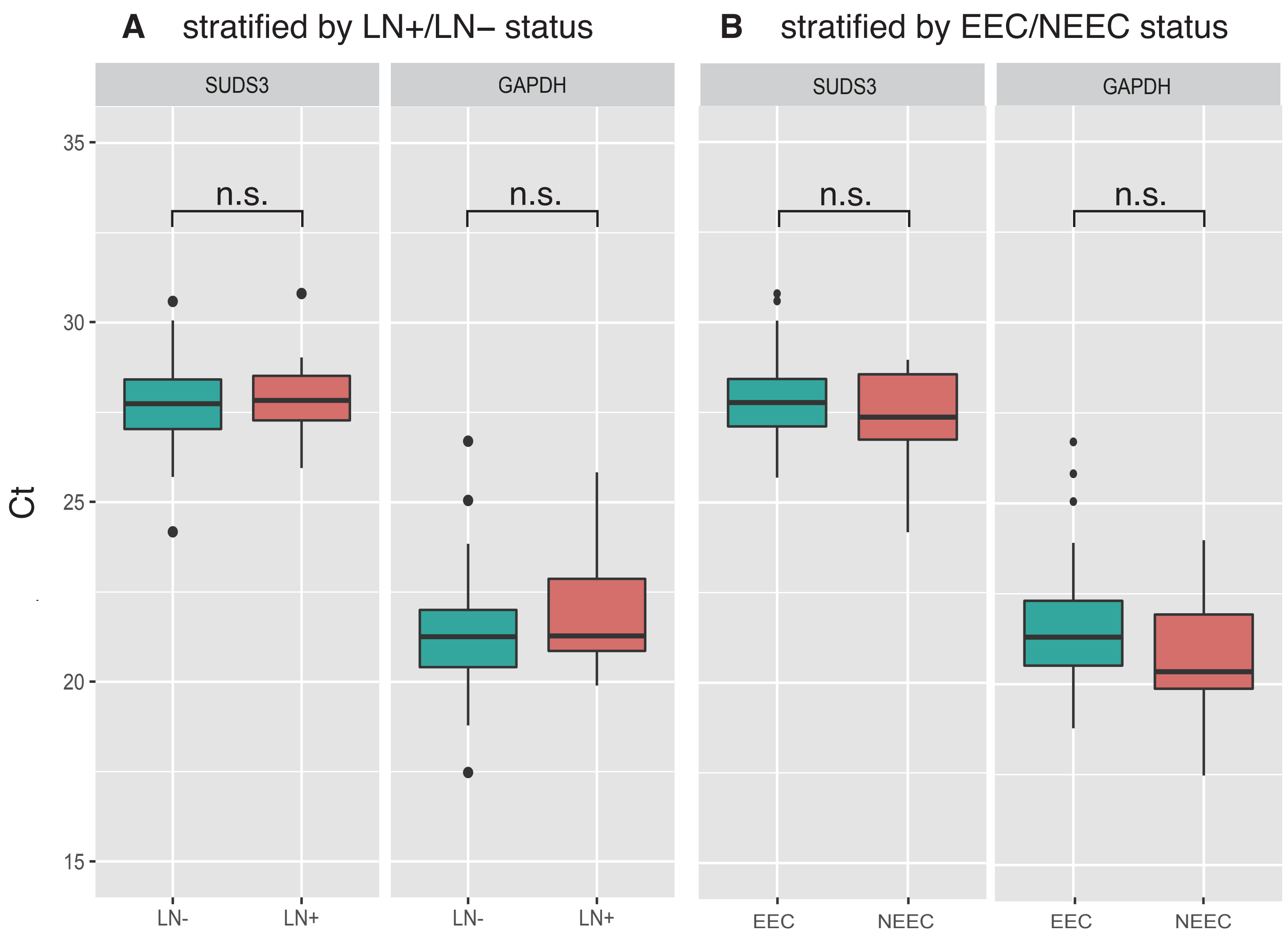

Supplementary Figure 5: Comparison of control gene expression in 115 endometrial cancer tissue samples. n.s., not significant. Box plots of SUDS3 mRNA expression and GAPDH mRNA expression stratified by LN+/LN– status (A) and endometrioid endometrial cancer (EEC)/non-endometrioid endometrial cancer (NEEC) status (B). There were no significant differences in control gene expression (A; *SUDS3*, $P = 0.5055$; *GAPDH*, $P = 0.2660$, B; *SUDS3*, $P = 0.5210$; *GAPDH*, $P = 0.1982$). The standard deviation of *SUDS3* was lower than that of *GAPDH* (*SUDS3*, SD = 1.012; *GAPDH*, SD = 1.464). The Ct mean of *SUDS3* was 27.74 and that of *GAPDH* was 21.37. The stability of *SUDS3* expression in the 115 endometrial cancer tissues was confirmed by qRT-PCR.

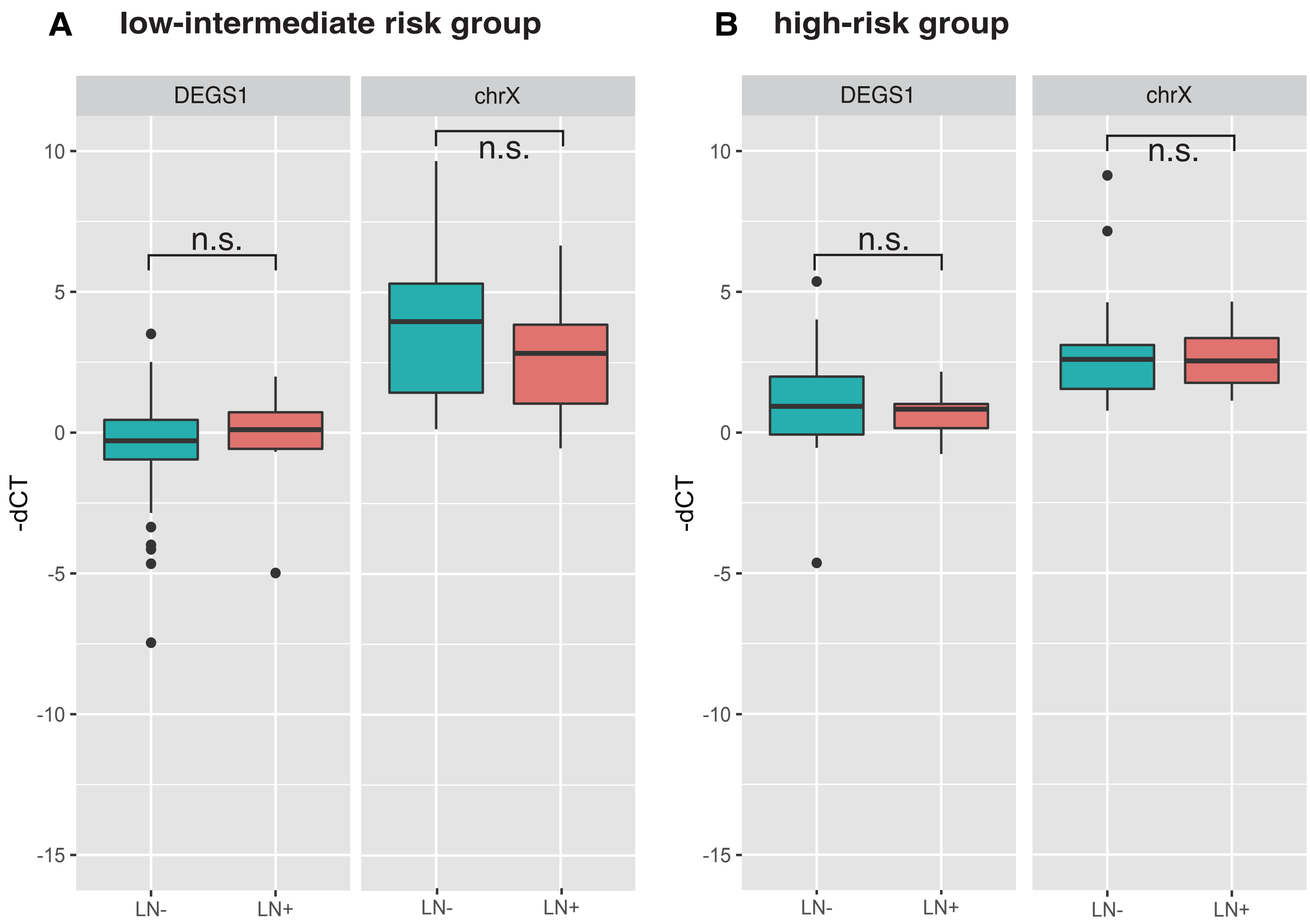

Supplementary Figure 6: Box plot of *DEGS1* mRNA and *chrx* mRNA.

DEGS1 mRNA and chrx mRNA expression was not significantly different between LN– and LN+ cases. However, the DEGS1 primer we used was designed for a common sequence of its variants. It has no specificity to capture p3@DEGS1 only. Therefore, there is a possibility that the p3 @ DEGS1 specific primer can significantly different between LN– and LN+ cases.

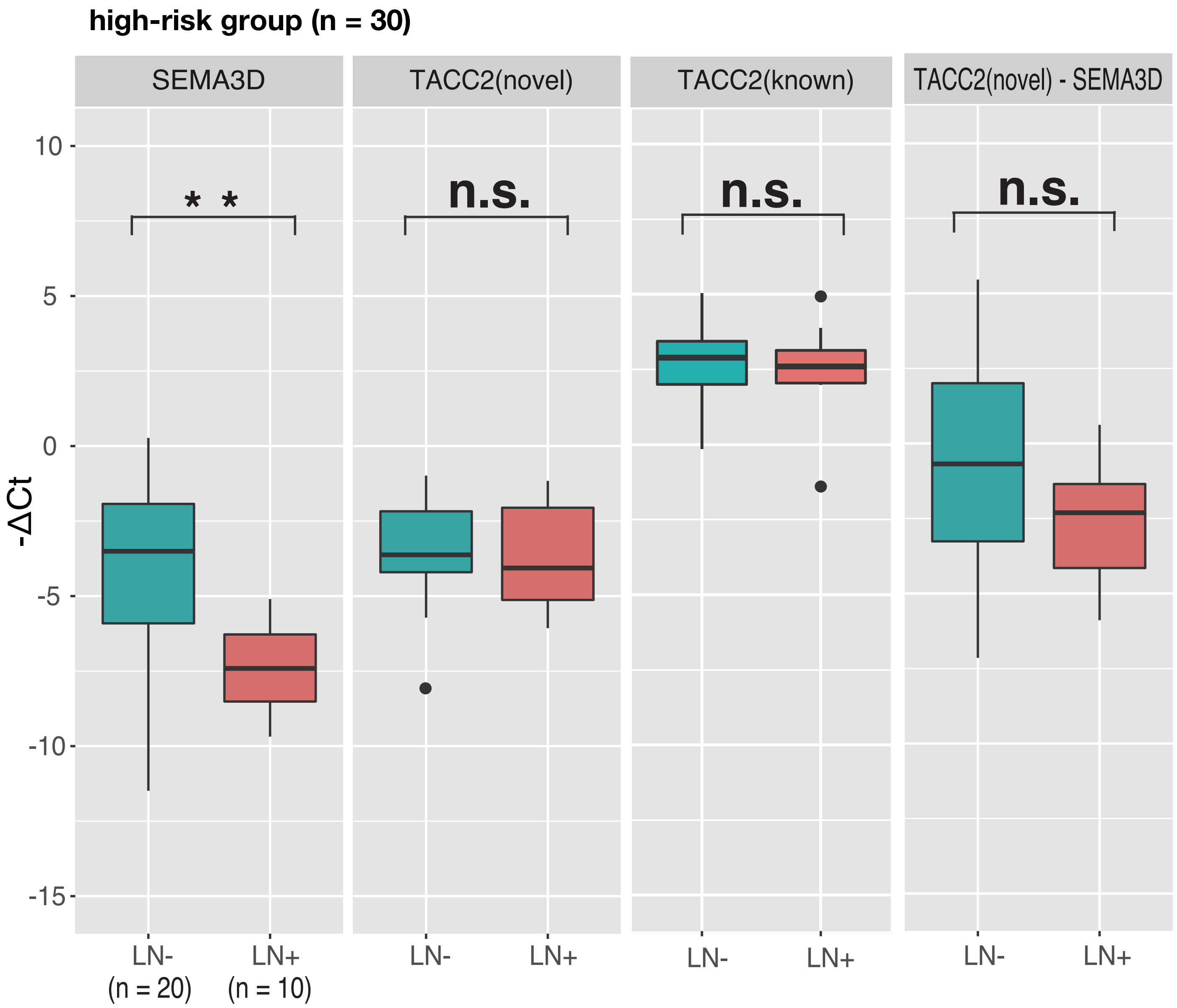

Supplementary Figure 7: Box plot of *SEMA3D* mRNA and novel *TACC2* mRNA in high-risk group.

**, $P < 0.01$; *, $P < 0.05$; n.s., not significant.

SEMA3D mRNA expression was significantly different between LN– and LN+ cases ($P = 0.0017$).

However, neither novel nor known *TACC2* mRNA expression was significantly different between LN– and LN+ cases.

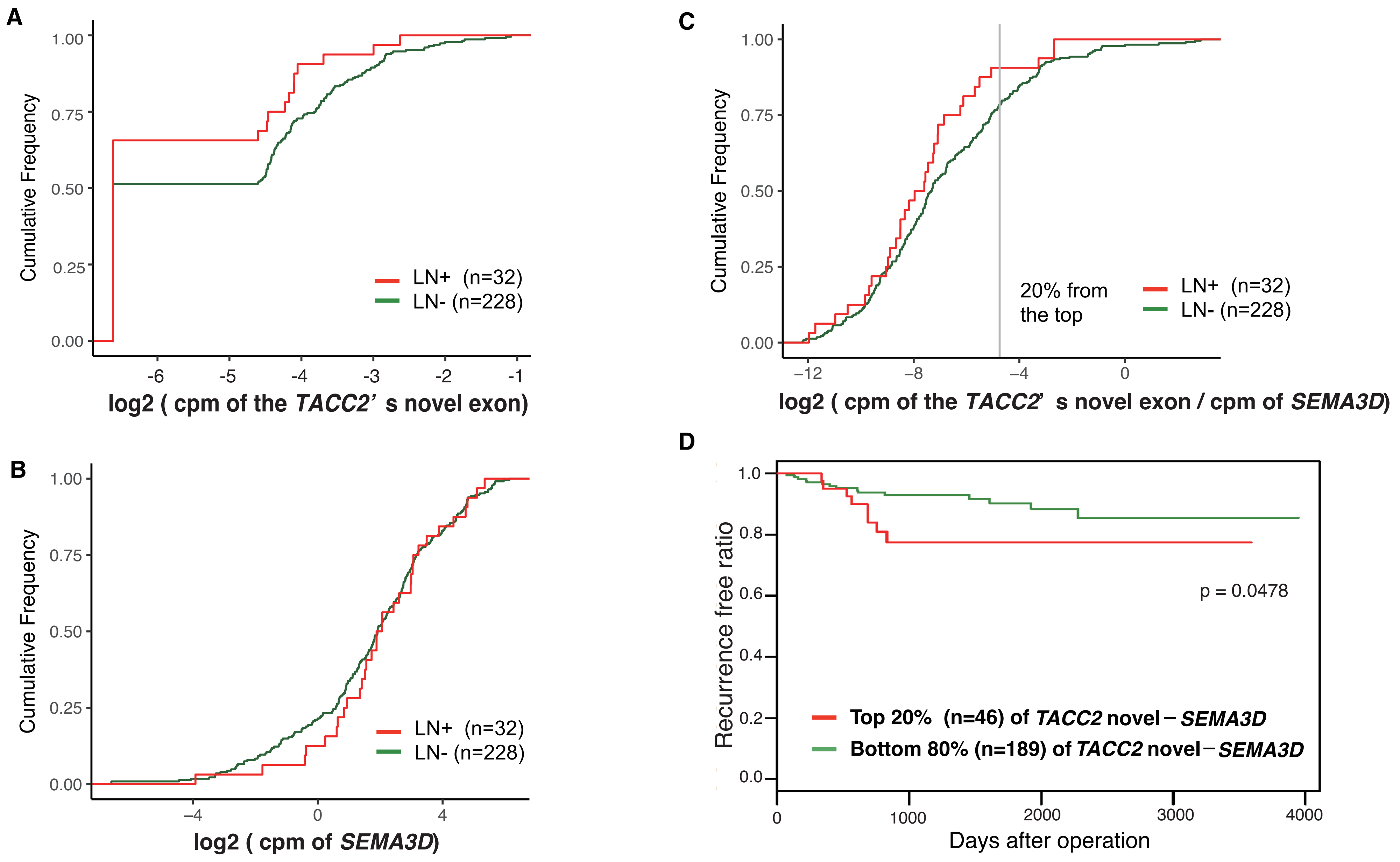

Supplementary Figure 8. Analysis of novel *TACC2* isoforms and *SEMA3D* in the TCGA Uterine Corpus Endometrial Carcinoma dataset

Of 548 patients with uterine corpus endometrial carcinoma in TCGA, 260 with endometrioid adenocarcinoma without clinical stage II, IIB, IIIA, IIIB or IV profiled by single-end RNA-seq were chosen after excluding one patient whose clinical stage did not match the number of lymph nodes with metastases. Cumulative distributions of the expression levels are shown for the TACC2's novel exon (A) and SEMA3D (B). Cumulative distribution for their expression difference is shown in (C), where its top 20% is indicated by gray line. Of the 260 patients, 235 with recurrence free survival data were subjected to Kaplan–Meier analysis based on the group of the top 20% of the expression difference and the rest (D).

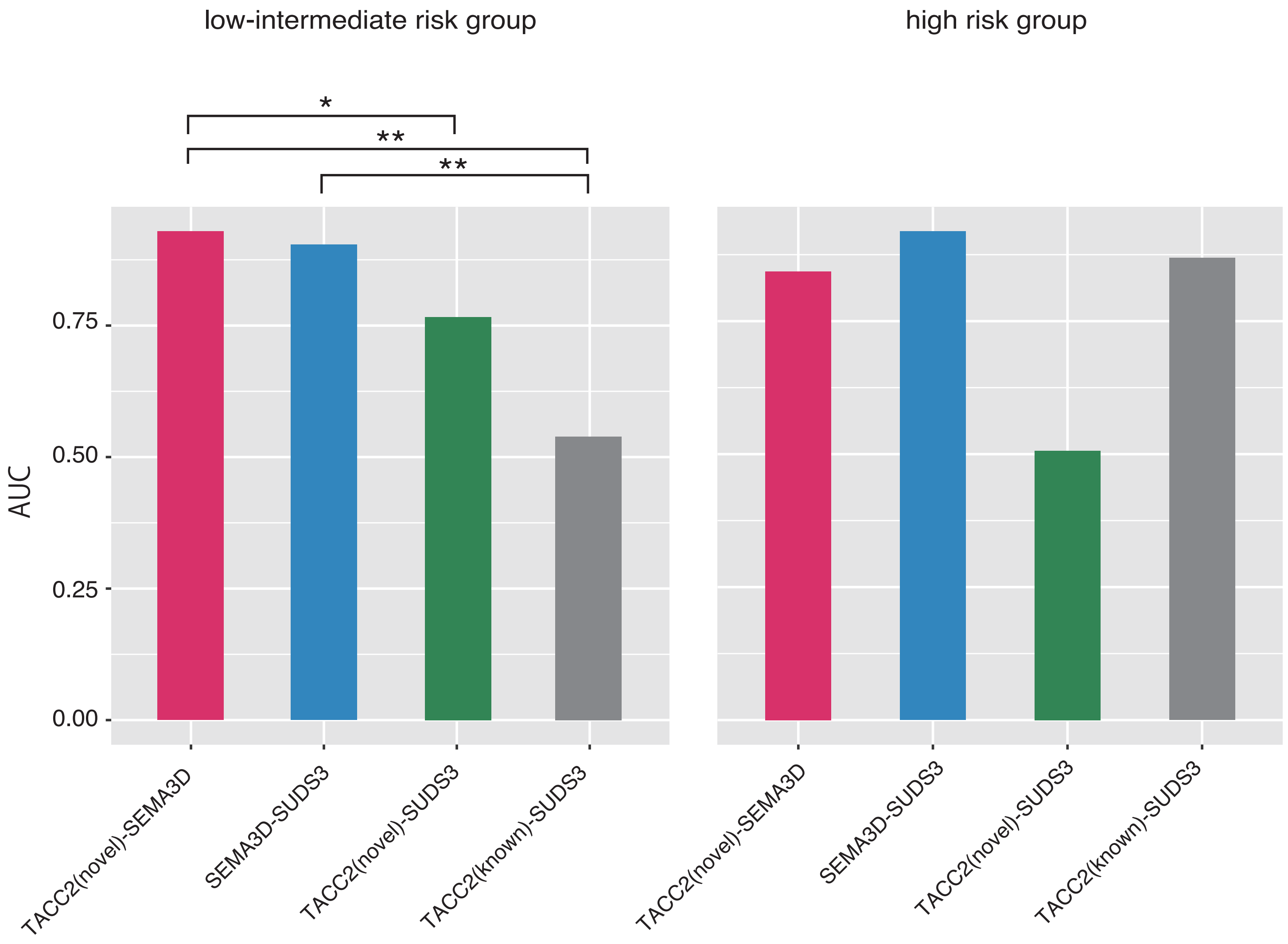

Supplementary Figure 9. Comparison of AUCs in each risk group using the bootstrap method.

**, P < 0.01; *, P < 0.05.

The AUC based on the expression difference between *TACC2* (novel) and *SEMA3D* was slightly increased to 0.929 compared to that for the expression difference between *SEMA3D* and SUDS3; however, the increase was not statistically significant. These results indicate the contribution of SEMA3D as the most substantial, whereas the novel isoform of *TACC2* is effective for confirming the results obtained with *SEMA3D*.

Supplementary Table 1. Preoperative feature-based risk groups.

	Histology					
	EM Ca G1 or G2		EM Ca G3		Other Pathology	
Spread of primary tumor	LN-	LN+	LN-	LN+	LN-	LN+
Myometorial invasion ≤ 1/2	57*	5*	4*	0*	3	0
Myometorial invasion > 1/2	16*	3*	2	2	2	2
Cervical invasion	5	1	1	0	0	0
Extra uterine	3	5	0	0	4	0

NOTE: Low-intermediate risk group were combined to form one category, and we devided all the patient into two groups: low-intermediate risk group(*) , and high risk group(no mark).

Supplementary Table 2. Six metastatic marker candidates.

Name	log (Fold Change)	log (CPM)	PValue	FDR	AUC
chr6,18277503,18277537,+;p@chr6:18277503..18277537,+	-6.14	0.48	2.67E-15	3.28E-11	1
chr7,84816122,84816189,-;p1@SEMA3D	6.86	3.24	2.65E-07	8.25E-05	1
chr1,224370589,224370611,+;p3@DEGS1	-5.91	2.66	1.60E-17	3.94E-13	1
chrX,23710342,23710348,-;p@chrX:23710342..23710348,-	-8.31	1.34	4.69E-16	7.69E-12	1
chr10,123779269,123779314,+;p10@TACC2	-4.08	0.99	7.85E-08	3.58E-05	0.98
chr8,9009045,9009059,-;p4@PPP1R3B	-4.04	1.81	4.04E-09	4.52E-06	0.98

Supplementary Table 3. Primers for long PCR and primer walk sequence of novel *TACC2* mRNA isoforms.

Primers for long PCR	Sequence
PF	5'-CCAGTTGCTGAAGGGCAGAA-3'
PR	5'-TTGCCTCGAACCTGAGCAATC-3'

Primers for primer walk sequence	Sequence
M13	5'-GTAAAACGACGGCCAGT-3'
P1	5'-CCTGGATCCATGCCTTGTGT-3'
P2	5'-AGAGAGCCAATGAAGGCACC-3'
P3	5'-CAGAGAAAGAGGCCAAGGGG-3'
P4	5'-GGAGAGCACTTGAACACGGA-3'
P5	5'-ATCTGGCCTACCACACAAGC-3'
P6	5'-GAGAACTTGGCAGCAGACCT-3'
P7	5'-GTCAGCACCAACGAGACAGA-3'
P8	5'-ATGAAACCCAGGAAGGCAGG-3'
P9	5'-GCCTCCTTGAAGCTGTTTGC-3'
P10	5'-GGGTAAAGACCTCACCAGGC-3'
P11	5'-GGAGTCACTGCTGGCATCTT-3'
P12	5'-ACGAAACTCACGACCCGAAG-3'
P13	5'-GAGTCAACGACCCCTGTCAAA-3'
P14	5'-CGTGCCTCAGACGCTAAGAA-3'
P15	5'-CTTCTGGAGGTGGCAGAGTG-3'
P16	5'-TCCTCTAAGACCCCCAGCTC-3'
M13 rev.	5'-CAGGAAACAGCTATGAC-3'

Supplementary Table 4: Number of CAGE reads representing promoter activities.

Donor	Metastasis	Reads from promoter	Reads from other regions	Total mapped reads	Promoter ratio	Comment
A	LN+	4,796,793	1,821,425	6,618,218	72%	
B	LN+	9,477,740	2,328,104	11,805,844	80%	
C	LN+	3,395,328	3,406,886	6,802,214	50%	
D	LN+	2,539,245	1,177,135	3,716,380	68%	
E	LN+	1,417,385	863,171	2,280,556	62%	not used for data analysis
F	LN−	4,948,430	1,227,079	6,175,509	80%	
G	LN−	4,725,262	2,055,504	6,780,766	70%	
H	LN−	6,917,926	2,341,863	9,259,789	75%	
I	LN−	6,545,061	1,701,530	8,246,591	79%	
J	LN−	5,870,606	2,565,676	8,436,282	70%	
K	LN−	5,365,102	2,118,532	7,483,634	72%	
L	LN−	6,969,970	1,954,930	8,924,900	78%	
M	LN−	6,136,067	2,534,944	8,671,011	71%	
N	LN−	7,144,001	1,524,665	8,668,666	82%	
O	LN−	9,638,949	2,221,999	11,860,948	81%	
Average				7,715,421	73%	

Supplementary Table 5. Comparison of performance across the methods for discrimination of LN+/LN– status in case of low-intermediare risk of recurrence

Biomarker based on the expression difference between SEMA3D and the novel TACC2 isoforms	Low-intermediare risk of recurrence		
	LN+	LN-	total
Metastasis positive	8	10	18
Metastasis negative	0	67	67
Total	8	77	85

Sensitivity 100%
Specificity 87.0%
Positive Predictive Value, PPV 44.4%
Negative Predictive Value, NPV 100%

Preoperative imaging diagnosis	Low-intermediare risk of recurrence		
	LN+	LN-	total
Metastasis positive	3	4	7
Metastasis negative	5	73	78
Total	8	77	85

Sensitivity 37.5%
Specificity 94.8%
Positive Predictive Value, PPV 42.8%
Negative Predictive Value, NPV 93.5%
